# Secreted Frizzled-Related Protein 1 as a Biomarker against Incomplete Age-Related Lobular Involution and Microcalcifications’ Development

**DOI:** 10.3390/cancers12092693

**Published:** 2020-09-21

**Authors:** Alisson Clemenceau, Mirette Hanna, Kaoutar Ennour-Idrissi, Anna Burguin, Caroline Diorio, Francine Durocher

**Affiliations:** 1 Department of Molecular Medicine, Faculty of Medicine, Laval University, Quebec, QC G1V 0A6, Canada; alisson.clemenceau@crchudequebec.ulaval.ca (A.C.); anna.burguin@crchudequebec.ulaval.ca (A.B.); 2 Cancer Research Centre, CHU de Quebec Research Centre, Quebec, QC G1V 4G2, Canada; mirette.hanna.1@ulaval.ca (M.H.); kaoutar.ennour-idrissi.1@ulaval.ca (K.E.-I.); caroline.diorio@crchudequebec.ulaval.ca (C.D.); 3 Department of Preventive and Social Medicine, Faculty of Medicine, Laval University, Quebec, QC G1V 0A6, Canada

**Keywords:** breast, breast cancer, secreted frizzled-related protein 1, SFRP1, lobular involution, breast involution, microcalcifications, inflammation, apoptosis, adipogenesis

## Abstract

**Simple Summary:**

Age-related lobular involution, a physiological breast atrophy, is a natural weapon against breast cancer development. Microcalcifications, which are present in 90% of diagnosed ductal carcinoma in situ, are associated with tumor invasiveness and aggressivity, but their impact on lobular involution remains unknown. In this study, we highlighted two predictive models for incomplete lobular involution and microcalcifications development. By identifying women at high risk of incomplete lobular involution, we hope to better prevent breast cancer development and so, to reduce late diagnostics. By preventing microcalcifications development, we will offer a new preventive tool for women younger than 50 years old who do not have access to mammography. We also identified two distinct inflammatory profiles associated with age-related lobular involution in parous and nulliparous women. These results warrant further investigation and could be crucial in personalized therapy development against breast cancer.

**Abstract:**

As a downregulator of the Wnt signaling pathway, SFRP1 is involved in several components of the age-related lobular involution process such as inflammation, apoptosis, and adipogenesis. Because microcalcifications are associated with inflammation, we aimed to demystify the cross talk between SFRP1, inflammatory markers, and microcalcifications by assessing SFRP1 expression (immunohistochemistry) in a cohort of 162 women with different degrees of lobular involution. SFRP1 expression was inversely associated with the degree of lobular involution (OR = 0.84; *p*-value < 0.01). SFRP1 expression, age at mastectomy, and waist circumference taken together predicted the degree of lobular involution (AUC = 78.1). This predictive model was best in patients with microcalcifications (AUC = 81.1) and in parous women (AUC = 87.8). SFRP1 expression was correlated with leptin (rho = 0.32), TNF-α (rho = 0.21), and IL-6 (rho = 0.21) expression by epithelial cells (all *p*-values <0.001). SFRP1 expression was lower in nulliparous women with involuted breast tissue compared with parous women with involuted breast tissue (Δmean = −2.31; *p*-value < 0.01) and was higher in nulliparous women with microcalcifications compared with nulliparous women without microcalcifications (Δmean = 2.4; *p*-value < 0.05). In this study, we highlighted two SFRP1-based predictive models for incomplete lobular involution and the development of microcalcifications and identified two distinct inflammatory profiles associated with age-related lobular involution in parous and nulliparous women.

## 1. Introduction

Secreted frizzled-related protein 1 (SFRP1) is a 314 amino acid protein composed of three domains: A peptide signal (PS), a frizzled (FRI), and an extracellular cysteine-rich domain (CRD) largely conserved between species [1]. It is a Wnt signaling pathway antagonist secreted by epithelial cells [2]. SFRP1 binds both Wnt proteins and the frizzled domain of fixation of Wnt proteins by homology. Hence, SFRP1 is involved in the downregulation of both canonical and noncanonical Wnt signaling pathways [2,3,4,5]. Because of the crucial role of Wnt signaling pathway in embryonic development and in regulation of tissue homeostasis, SFRP1 dysregulation is largely associated with cancer development, including breast cancer [6,7]. The biological mechanisms explaining these associations remain poorly understood.

SFRP1 seems to be a crucial player in all steps of the age-related lobular involution process [8,9,10]. Age-related lobular involution, a physiological breast atrophy described as a natural protective process against breast tumorigenesis [11,12,13], is composed of three known phenomena: Inflammation, apoptosis, and adipogenesis [14,15]. SFRP1 plays a role in each of these physiological phenomena independently, yet it has never been directly associated with breast involution. In fact, *SFRP1* is upregulated in physiological inflammatory conditions [16,17]. Furthermore, *SFRP1* downregulation in breast tissue is associated with negative regulation of the transcription of apoptotic genes [18,19], which are absolutely needed for complete lobular involution. To complete the loop, SFRP1 is upregulated in early adipogenesis because of its role in promoting pre-adipocytes’ maturation [20]. When SFRP1 is lacking, pre-adipocytes, which are unable to mature, produce an excess of adipokines and chemokines that exacerbate inflammation. However, chronic inflammation is inversely proportional to lobular involution [15]. Furthermore, SFRP1 is involved in the positive regulation of apoptosis of mammary epithelial cells [18,19]. That means that the lack of SFRP1 in breast tissue could result both in incomplete lobular involution and in breast hyperplasia. The microenvironment is also known to be a crucial player in tumor development. Another protagonist is possibly involved in the chronic inflammation of breast tissue. Microcalcifications, present in 30 to 50% of all malignant breast lesions, are effectively associated with protumorigenic inflammatory cytokines such as interleukine-8 (IL-8) [21].

We hypothesized that SFRP1 underexpression during perimenopausal lobular involution is responsible for the accumulation of microcalcifications in breast tissue. We also postulated that epithelial cells’ replacement by adipose tissue increases chronic inflammation and the risk of development of cancer due to the presence of immature cells. To demystify the interconnection between all these protagonists, we compared SFRP1 expression profile with the degree of lobular involution and the presence of microcalcifications in nontumoral breast tissue of 162 breast cancer patients. Moreover, to better differentiate physiological inflammation from pathologic inflammation, we also tested the association of previously cited variables with multiple inflammatory markers already known to be modulated during lobular involution [15].

## 2. Results

### 2.1. Characteristics of the Study Population

Characteristics of the study population are presented in Table 1. The average age at mastectomy in our population was 52.4 ± 7.9 years old. As expected, age at mastectomy was higher in postmenopausal women (58.3 ± 4.9 years) compared with premenopausal women (46.7 ± 5.8 years; *p*-value < 0.001). Because of this relationship between age and menopause, we observed more involuted breast tissue among postmenopausal women (*n* = 54; 68%) compared with premenopausal women (*n* = 30; 38%; *p*-value < 0.0001). Complete involution is characterized by predominant type 1 lobules in the absence of type 3 lobules. Type 3 lobules are the result of the addition of mammary branchings during puberty and pregnancy. Because postmenopausal women sustain postlactational lobular involution in addition to age-related lobular involution around the age of 40, we observed the greatest proportion of type 1 lobules in postmenopausal (*n* = 49; 61%) compared to premenopausal women (*n* = 16; 20%; *p*-value < 0.0001). However, the distribution of women according to number of deliveries, age at first delivery, or average duration of breastfeeding was not significantly different between premenopausal and postmenopausal women. Because SFRP1 is related to adipogenesis, and in the hope to segregate menopause-related adipogenesis to lobular involution-related adipogenesis, we also paid particular attention to waist circumference, a marker of adiposity, to distinguish menopause-related adipogenesis from lobular involution-related adipogenesis. As expected, waist circumference was higher in postmenopausal women (90.1 ± 12.5 cm) compared with premenopausal women (83.9 ± 12.3 cm; *p*-value < 0.01). Finally, to test the hypothesis of a switch of SFRP1 expression related to the breast microenvironment, we analyzed the presence of microcalcifications according to menopausal status. No significant difference between both groups was observed.

### 2.2. Association between SFRP1 Expression and the Degree of Lobular Involution

#### 2.2.1. Immunohistochemistry (IHC)

**Figure 1:**
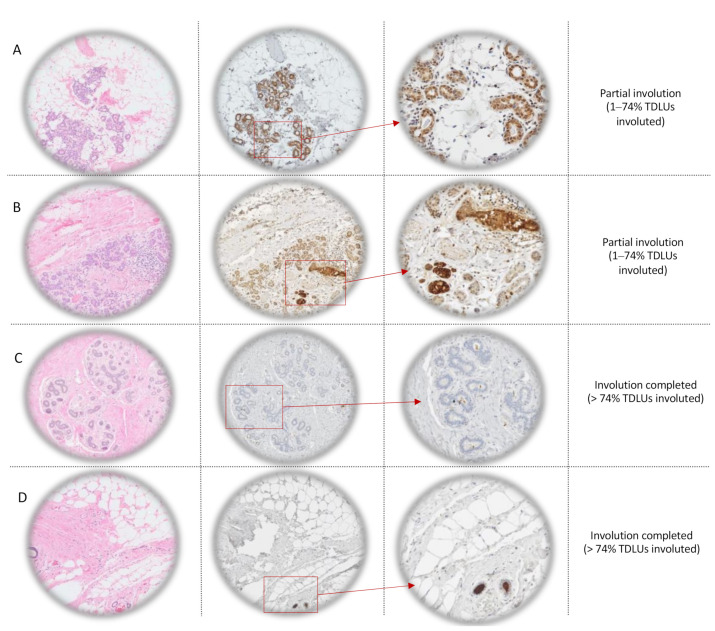
Lobule types’ description and SFRP1 immunohistochemistry scores’ explanation. Column 1: Hematoxylin-eosin (H&E) staining of nontumoral tissue at different degrees of lobular involution (rows **A** and **B**: Partially involuted (1–74% TDLUs involuted) breast tissue obtained from premenopausal women without (row **A**) and with (row **B**) microcalcifications and rows **C** and **D**: Completely involuted (>74% TDLUs involuted) breast tissue obtained from premenopausal women with microcalcifications at light microscope ×5). Columns 2 and 3: Immunohistochemistry against SFRP1 (row **A**: Score 2; row **B**: Score 6, row **C**: Score 0; and row **D**: Score 1) at light microscope ×5 (column 2) and ×20 (column 3). TDLUs = terminal duct lobular units.

Types 2–3 lobules in partially involuted breast tissue (1–74% involuted terminal duct lobular units (TDLUs)) are illustrated in Figure 1A,B along with SFRP1 immunostaining. Sample illustrated in Figure 1A came from a premenopausal woman without microcalcification while sample illustrated in Figure 1B came from a premenopausal woman with microcalcifications. In both cases, lobules are characterized by numerous acini, resulting from mammary gland branching during puberty and pregnancy. Cytoplasm and luminal plasma membrane of epithelial cells are positive, suggesting secretion of SFRP1 by epithelial cells. We also observed the presence of cells from the immune system around the positive lobules. In Figure 1C, we observed negative type 2 lobules, in complete involuted breast tissue (>74% TDLUs involuted) obtained from a premenopausal woman with microcalcifications. In Figure 1D, also obtained from a premenopausal woman with microcalcifications, we observed type 1 lobules, which are defined as lobules with less than 12 acini. They did not display SFRP1 expression in the epithelial cells.

#### 2.2.2. Association Study

SFRP1 expression in nontumoral tissue of all women (*n* = 162) was associated with the degree of age-related lobular involution before (odds ratio (OR) = 0.85; 95% confidence interval (CI) = 0.76–0.95; *p*-value < 0.01; Table 2) and after adjustment for age at mastectomy (OR = 0.84; 95% CI = 0.74–0.94; *p*-value < 0.01), which is a causal variable of involution (OR = 1.11; 95% CI = 1.06–1.16; *p*-value < 0.0001). In fact, SFRP1 was expressed in non-involuted tissue, while it was lower when lobular involution was complete. Stratification by menopausal status did not change the association of SFRP1 with age-related lobular involution (premenopausal: OR = 0.81; 95% CI = 0.66–0.98; *p*-value < 0.05; postmenopausal: OR = 0.83; 95% CI = 0.7–0.98); *p*-value < 0.05).

**Figure 2:**
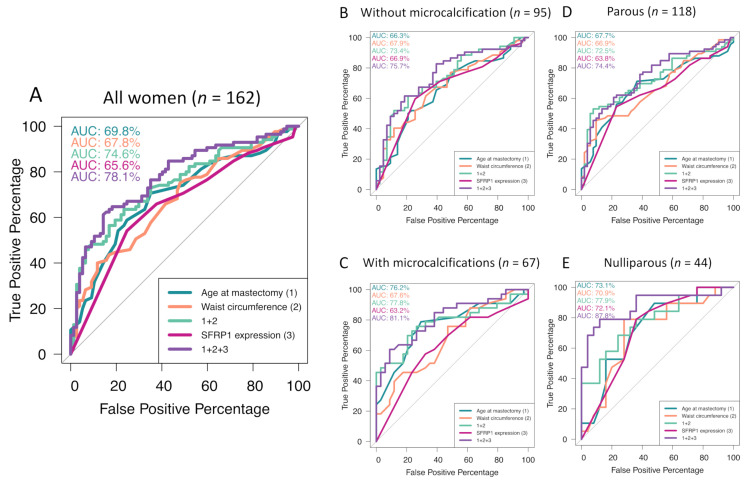
Performance study of age-related lobular involution predictive models by ROC curves’ schematization. Age-related lobular involution predictive model for all women (**A**), women without microcalcifications (**B**), having microcalcifications (**C**), parous women, and (**D**) nulliparous women (**E**). ROC = receiver operating characteristic, AUC = area under the curve.

Because age and menopausal status are correlated, we tested the model with menopausal status instead of age at mastectomy. As observed with age, menopausal status had no impact on the association between SFRP1 expression and the degree of lobular involution (OR = 0.84; 95% CI = 0.74–0.95; *p*-value < 0.01). To challenge the robustness of SFRP1 association with the degree of age-related lobular involution, we used different breast microenvironment characteristics to adjust our models (Table 2). The presence of microcalcifications (OR = 0.93; 95% CI = 0.45–1.94; *p*-value = 0.85) was not associated with the degree of lobular involution and had no impact on SFRP1 association with breast involution in all women (OR = 0.85; 95% CI = 0.75–0.95; *p*-value < 0.01). However, waist circumference was strongly associated with the degree of lobular involution (OR = 1.06; 95% CI = 1.03–1.09; *p*-value < 0.0001) but without any impact on the association of SFRP1 expression with the degree of lobular involution in all women (OR = 0.84; 95% CI = 0.74–0.94). When considered together in the same model, age at mastectomy (OR = 1.09; 95% CI = 1.04–1.15; *p*-value < 0.001), the presence of microcalcifications (OR = 0.93; 95% CI = 0.45–1.94; *p*-value = 0.85), and waist circumference (OR = 1.05; 95% CI = 1.02–1.09; *p*-value < 0.01) had no impact on the association between SFRP1 expression and the degree of lobular involution in all women (OR = 0.83; 95% CI = 0.73–0.94; *p*-value < 0.01). When SFRP1 expression was considered as a binary variable (any positive staining vs. the total absence of staining), the results followed the same trend (Table S1). To evaluate the predictive value of SFRP1 expression for predicting physiological complete involution, which is a protective factor against breast tumorigenesis, we performed receiver operating characteristics (ROC) curves (Figure 2). The area under the curves (AUC) indicated that SFRP1 expression improves the predictive power (AUC = 78.1) of a model including both age and waist circumference in all women (AUC = 74.6; Figure 2A). Interestingly, when stratified according to the presence or absence of microcalcifications, the AUC for the same model was improved to 81.1% (Figure 2B,C).

#### 2.2.3. Inflammatory Profile

To better understand the biological mechanism involved in SFRP1-related lobular involution, we performed Spearman correlations between SFRP1 expression and cytokines, or chemokines known to regulate the involution-related inflammation [15] (Table 3). SFRP1 expression was positively correlated with leptin (rho = 0.32; *p*-value < 0.0001), tumor necrosis factor α (TNF-α; rho = 0.21; *p*-value < 0.001) and interleukin 6 (IL-6; rho = 0.21; *p*-value < 0.001) expression by epithelial cells in all women. SFRP1 expression also tended to be positively correlated with C-reactive protein expression (CRP; rho = 0.14; *p*-value = 0.09) and serum amyloid A-1 protein (SAA1; rho = 0.15; *p*-value = 0.07) expression by epithelial cells. Leptin is a cytokine produced during adipogenesis by both epithelial cells and adipocytes. In our cohort, expression of leptin by epithelial cells was negatively correlated with waist circumference (rho = −0.16; *p*-value < 0.05). However, leptin expression by adipocytes was strongly correlated with waist circumference (rho = 0.45; *p*-value < 0.0001). The same trend was observed for TNF-α (rho = 0.16; *p*-value = 0.21) and IL-6 (rho = 0.37; *p*-value < 0.01) expression by epithelial cells. However, leptin (rho = 0.043; *p*-value = 0.72), TNF-α (rho = −0.078; *p*-value = 0.54), and IL-6 (rho = 0.041; *p*-value = 0.73) expression by adipocytes was not correlated with SFRP1 expression in all women. These results suggest a switch of leptin, TNF-α, and IL-6 production between epithelial cells and adipocytes during breast involution-related adipogenesis. However, CRP expression by epithelial cells (rho = −0.22; *p*-value < 0.01) was significantly correlated with waist circumference without being correlated with SFRP1 expression (rho = 0.14; *p*-value = 0.09).

#### 2.2.4. Reproductive History

To better identify the origin of inflammatory molecules and to characterize the inflammatory process during involution, we stratified our cohort according to parity status (Table 4). The lower expression of leptin (Δmean = −2.53; *p*-value < 0.001), TNF-α (Δmean = −2.15; *p*-value < 0.01), and IL-6 (Δmean = −2.42; *p*-value < 0.01) in involuted breast tissue seemed nonimpacted by parity history. SAA1 was lower in nulliparous women having involuted breast tissue (Δmean = −0.94; *p*-value < 0.05). On the other hand, CRP and IL-10 expression seemed lower in involuted breast tissue compared with non-involuted breast tissue, independently from parity history. As for lactoferrin, its expression was negatively associated with breast tissue involution in nulliparous women (Δmean = −2.31; *p*-value < 0.01) compared with non-involuted breast tissue. These results suggest that the first lobular involution, which corresponded to the age-related lobular involution in nulliparous women, was different from the following ones. These results, combined with those presented in Table 3, suggest that leptin, TNF-α, IL-6, and CRP are involved in breast involution-related adipogenesis potentially initiated by SFRP1 expression. SAA1 may be involved in breast involution due to SFRP1 but in nulliparous women only, IL-10 may be involved in breast involution independently of SFRP1 expression, and adipogenesis and lactoferrin were lower in nulliparous women having involuted breast tissue. Finally, we observed that SFRP1 expression in nulliparous women was dramatically lower in involuted breast tissue compared with non-involuted breast tissue (*p*-value < 0.01), but also in comparison with parous women having involuted breast tissue. To further understand this unexpected result, we performed ROC curves to test the value of SFRP1 expression as a predictive tool for the degree of lobular involution, by stratifying the cohort by parity status. In parous women (Figure 2D), SFRP1 expression improved the model including age at mastectomy and waist circumference alone (AUC = 72.5). The result was noticeable in nulliparous women (Figure 2E). SFRP1 expression alone was a good predictor of lobular involution (AUC = 72.1), but when added to the model, including age at mastectomy and waist circumference variables, the AUC reached 87.8 compared to 77.9 when age and waist circumference were considered alone. 

### 2.3. Association between SFRP1 and the Presence of Microcalcifications

#### 2.3.1. Association Study

**Figure 3:**
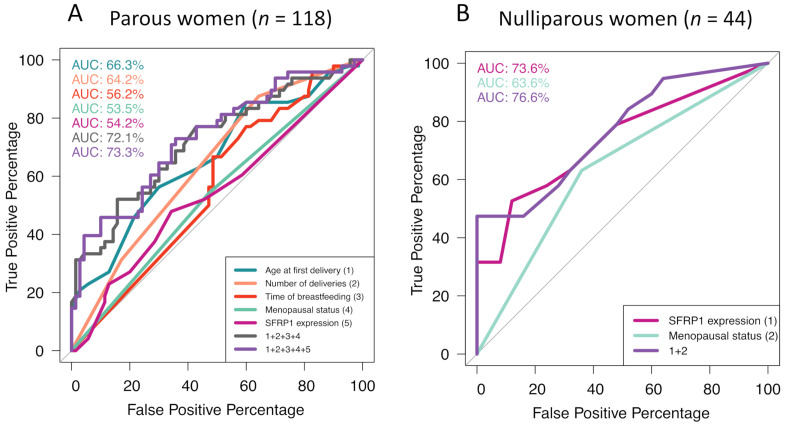
Performance study of microcalcifications’ development predictive models by ROC curves’ schematization. Microcalcifications’ development predictive model in parous women (**A**) and nulliparous women (**B**). ROC = receiver operating characteristic, AUC = area under the curve.

Because the presence of microcalcifications is symptomatic of inflammatory disorders in soft tissues [21], we explored the potential cross-link between the molecular profile associated with lobular involution and the one associated with microcalcifications. Although the presence of microcalcifications was not associated with the degree of lobular involution in the overall population (OR = 0.95; 95% CI = 0.45–2; *p*-value = 0.89), it is important to highlight the difference of odds ratio after stratification by menopausal status. The presence of microcalcifications in premenopausal women tended to be associated with non-involuted mammary glands (OR = 0.48; 95% CI = 0.15–1.4; *p*-value = 0.19). However, the opposite was observed in postmenopausal women (OR = 2.29; 95% CI = 0.7–8.3; *p*-value = 0.18). To better understand the bioactivity of microcalcifications, we characterized the population according to the presence of such crystals in the breast tissue (Table 5). Age at first delivery in parous women was significantly associated with the development of microcalcifications (OR = 1.19; 95% CI = 1.07–1.34; *p*-value < 0.01). Having a first delivery before the age of 25 was protective, whereas after the age of 27 the risk of microcalcifications’ development was higher (*p*-value < 0.01). In addition, having three children or more (*p*-value < 0.01) and breastfeeding newborns for at least six months (*p*-value = 0.054) seemed to have a weak protective effect against breast microcalcifications’ development but this observation remains to be confirmed in other cohorts. On the other hand, SFRP1 expression was significantly associated with microcalcifications’ development in nulliparous women only (OR = 1.47; 95% CI = 1.14–2.04; *p*-value < 0.01). To test the accuracy of the previously cited variables in predicting the development of microcalcifications, we performed ROC curves (Figure 3). As expected, in parous women (Figure 3A), age at first delivery was the best predictive variable for breast calcifications (AUC = 66.3%). If SFRP1 expression was not a good variable to predict microcalcifications’ development in breast tissue when considered alone (AUC = 54.2%), it improved the model when added to age at first delivery, number of deliveries, length of breastfeeding, and menopausal status (AUC = 73.3%) compared with the last four variables alone (AUC = 72.1%). In nulliparous women (Figure 3B), SFRP1 expression alone was the best predictor of microcalcifications’ development (AUC = 73.6%). The model was improved by adding menopausal status (AUC = 76.6%).

#### 2.3.2. Inflammatory Profile and Reproductive History

Mean expression of inflammatory molecules in relation with the presence of microcalcifications are listed in Table 6. As previously described, SFRP1 expression was significantly higher in nulliparous women with microcalcifications compared with patients without microcalcifications (mean = 3.68 and 1.28 for nulliparous women with microcalcifications and without microcalcification, respectively, *p*-value < 0.05). This difference was also observed in parous women who did not breastfeed (Δmean = 1.57; *p*-value = 0.061). Furthermore, in presence of microcalcifications, the CRP expression in nulliparous women was decreased compared with patients without microcalcifications (Δmean = −1.36; *p*-value < 0.05). In addition, cyclooxygenase-2 (COX-2) expression in the stroma was lower in presence of microcalcifications (mean = 1.17) compared with the absence of microcalcifications (mean = 2.31; *p*-value < 0.01) in parous women who breastfed. Finally, transforming growth factor beta (TGF-β) expression in women who breastfed was significantly higher in presence of microcalcifications (mean = 2.79) compared with women without microcalcifications (mean = 1.29; *p*-value < 0.05). These results suggest the existence of different inflammatory profiles related to parity history and the presence of microcalcifications.

## 3. Discussion

**Figure 4:**
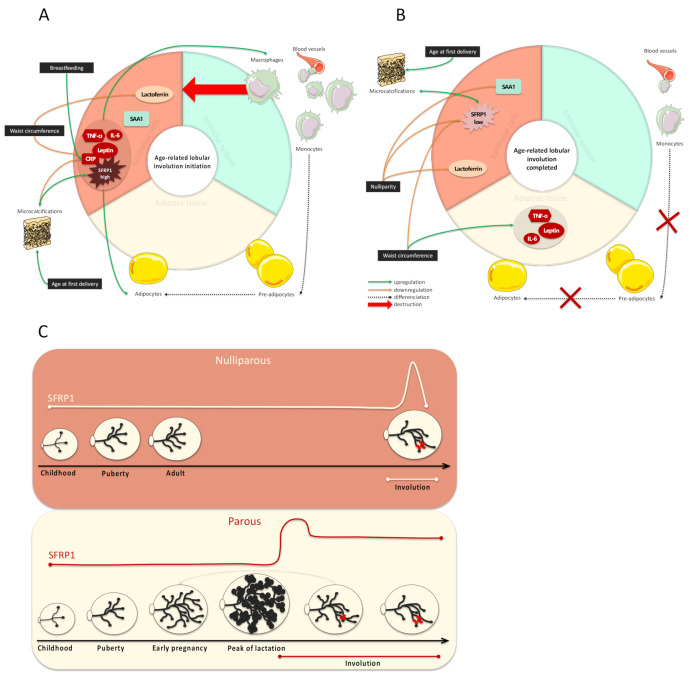
Schematic representation of the inflammatory profile at the initiation of the age-related lobular involution (**A**) and once it is completed (**B**) and SFRP1 expression modulation during a woman’s life considering the parity status (**C**). (**A**) SFRP1 induces the initiation of the age-related involution. Its expression is correlated with inflammatory molecules produced by epithelial cells (leptin, TNF-α, IL-6, and CRP) involved in the immune system activation and the involution-related adipogenesis. Microcalcifications’ development, which is exacerbated by advanced age at first delivery, is positively correlated with the expression of SFRP1 expression but negatively with CRP expression in nulliparous women specifically. (**B**) After lobular involution, SFRP1 is less expressed. Inflammatory molecules that were once produced by epithelial cells are now produced by adipocytes when lobular involution is completed. The absence of SFRP1 expression is also responsible for the decrease in adipogenesis, the downregulation of monocytes and pre-adipocytes’ differentiation into mature adipocytes and the decrease of the immune system activation. Lactoferrin is downregulated in nulliparous women. (**C**) The peak of SFRP1 expression occurs during age-related lobular involution in nulliparous women, resulting in a fast process followed by a dramatic fall in SFRP1 expression. On the other hand, the peak of SFRP1 expression occurs during the late phase of lactation in parous women, resulting in a progressive lobular involution followed by a slight decrease in the expression of SFRP1 expression.

A schematic representation of our results is presented in Figure 4. In this paper, we have shown that SFRP1 expression is associated with age-related lobular involution of breast tissue. However, when SFRP1 expression was analyzed according to both parity history and the degree of lobular involution, we observed that the differences of expression between involuted and non-involuted breast tissue were only present in women who did not breastfeed. This surprising result suggests that SFRP1 mediates the first lobular involution, which is a postlactation involution (Figure 4C). To our knowledge, this is the first study considering SFRP1 as a key player of age-related lobular involution in women. Because incomplete age-related lobular involution is associated with an increased risk of breast cancer development [11,12,13], it is essential to develop clinical tools to identify women at higher risk of incomplete lobular involution, and to increase their follow-up. 

This study is the first to consider lobular involution and microcalcifications’ bioactivity together to explain early breast carcinogenesis. We demonstrated that the expression of SFRP1 is higher in presence of microcalcifications. Hence, we also highlighted predictive models to predict microcalcifications’ development in both nulliparous and parous women. For parous women, a non-invasive tool to predict microcalcifications’ development could be evaluated in a clinical assay, without changing any clinical practices. In fact, the model includes parity and clinical information that could be obtained by performing interviews. Patients at high risk to develop microcalcifications could have more frequent follow-up visits to avoid late diagnosis and considerably improve breast cancer survival rates. This evidence is encouraging for the medical research community to explore *SFRP1* bioactivity and the lobular involution phenomenon. The absence of knowledge on both lobular involution and microcalcifications has led to an absence of consideration of this phenomenon on breast cancer treatment strategies. Furthermore, around 16% of breast cancers are diagnosed in women aged 30 to 49 years old, which represents the lobular involution time window. Unfortunately, women younger than 50 years old do not have access to mammography according to current prevention strategies. In fact, mammography testing is not efficient for breast cancer diagnosis in a high mammary density context. By developing the presented predictive tool against microcalcifications’ development, we offer an alternative for preventing breast cancer in women younger than 50 years old and in women with high mammary density. 

We also highlighted the existence of different inflammatory profiles related to breast involution, the microenvironment, and a woman’s parity history, emphasizing the complexity of breast tissue and the importance of personalized breast cancer treatments. In fact, the grey zone between inflammatory processes needed to initiate lobular involution and the pathological inflammation responsible for increased cell proliferation and migration is thin and remains misunderstood. SFRP1 expression was correlated with the expression of leptin, TNF-α, and IL-6, which are specifically involved in the age-related lobular involution adipogenesis, independently from a woman’s reproductive history (Figure 4A). Moreover, our model suggests that after involution, leptin, TNF-α, and IL-6 expression switch from epithelial cells to adipocytes (Figure 4B).

Interestingly, SFRP1 is described in the literature as an osteolytic protein. It is responsible for increased bone resorption in the bone tissue in both physiological and bone metastases from breast microenvironment [22,23]. As such, we observed that SFRP1 was correlated with leptin, IL-6, and TNF-α, three adipokines and cytokines involved in promoting bone resorption. In fact, leptin is known to induce IL-6 and TNF-α production, which is involved in osteoclastogenesis [24,25]. We can thus hypothesize that during SFRP1-mediated involution, the immature adipocytes in presence of microcalcifications develop an osteoblast-like activity responsible for the new microcalcifications’ production. In this condition, it is possible that SFRP1 function switches to engage in microcalcifications’ destruction, resulting in a pathological chronic inflammation responsible for the increased risk of breast cancer development. Obviously, these hypotheses will have to be tested in vitro.

The main limitation of our study resides in the fact that all our study participants were breast cancer patients. Hence, we cannot be certain that the molecular profile of the tissue was not already altered by the nearby breast cancer. However, we used normal breast tissue at least 1-cm distance from the tumor, and the inflammatory profile of the normal breast tissue adjacent to breast cancer was highly correlated with inflammatory profile of the healthy contralateral breast tissue [26]. Lastly, due to the cross-sectional design, we cannot infer a causal relationship from the observed associations.

## 4. Materials and Methods 

### 4.1. Study Population and Data Collection

Patients’ selection and data collection are already described in Hanna et al. [27]. Briefly, the study population included all women who received a unilateral breast cancer diagnosis before the age of 70 years old, at the Centre des Maladies du Sein in Quebec City (QC), Canada, between January 2011 and April 2012. These women had a partial or complete resection surgery (mastectomy) following diagnosis. Pregnant women as well as those who already had breast surgery or adjuvant therapy before diagnostic were not eligible. At the end of the recruitment period, 164 women satisfied the inclusion criteria and provided a signed and informed consent. The protocol was approved by the Research Ethics Board of the Centre Hospitalier Universitaire de Québec, Quebec City (QC), Canada (ethic code: DR-002-938). The histopathological characteristics of this consecutive series of women presenting with breast cancer were already detailed in Ennour-Idrissi et al. [28] (Table S2), and were very similar to those of the breast cancer population [29]. Anthropometric data were collected by nurses and reproductive data were collected during personal and telephone interviews. Women were included in the postmenopausal group if they had 12 months of amenorrhea whether naturally or induced. Women aged 53 or 55 years or more for nonsmokers and smokers, respectively, who had hysterectomy without bilateral oophorectomy were also considered as postmenopausal. Other women were included in the premenopausal group. The presence of microcalcifications was determined by reviewing pathology reports. 

### 4.2. Age-Related Lobular Involution Assessment

The degree of lobular involution was determined by two distinct pathologists on nontumoral breast tissue localized at least 1 cm from the tumor. This qualitative variable was established considering the percentage of TDLUs with no or few acini and no intralobular stroma. Women with a percentage of involuted TDLUs equal to or higher than 75% were considered as completely involuted, while women with less than 75% of involuted TDLUs in their breast tissue were considered as not or partially involuted (Figure 1). We also considered the proportion of each lobule type as a quantitative measure. Type 1 lobules are defined as lobules with less than 12 acini and types 2–3 lobules with 13 to 80 acini. Type 4 lobules are only present in pregnant women producing milk and eventually regress to types 2–3 lobules after stopping breastfeeding. The type of lobule representing more than 60% of all lobules observed was selected. Following the literature recommendations, the result of the tested breast was also admitted for the contralateral breast [30].

### 4.3. Anti-SFRP1 Immunohistochemistry Staining

Tissue microarrays (TMAs) were constructed with nontumoral TDLUs previously localized on the biopsy samples by two distinct pathologists at more than 1-cm distance from the tumor. Six cores, 1 mm in diameter for each of the 164 patients, were punched from the formalin-fixed paraffin embedded (FFPE) mastectomy blocks and randomly arrayed on virgin paraffin TMA blocks using the tissue punch arrayer (Beecher Instruments^®^ Tissue Microarray Technology, Estigen, Sun Prairie, WI, USA). Three serial slides of the final TMAs (4 µm thick) were cut. The first section was used for hematoxylin-eosin (H&E) staining and pathological examination, while the other two sections were used for immunohistochemistry (IHC) staining for SFRP1. As previously described in Burguin et al. [31], after samples’ dewaxing and rehydration, heat epitope antigen retrieval was performed during 30 min at 95.6 °C using prewarmed tris- ethylene glycol-bis(2-aminoethylether)-*N*,*N*,*N*′,*N*′-tetraacetic acid (EGTA) buffer (pH = 9). The endogen peroxidase was blocked using hydrogen peroxide. Nonspecific staining was also blocked (IDetect™ Super Stain System (HRP), Super Block). Then, slides were incubated with the primary antibody (Abcam; ab4193) at the optimal dilution (1:800) overnight at 4 °C in a wet chamber. The corresponding secondary antibody (Dako, EnVision™ + Dual Link System-HRP, Santa Clara, CA, USA) was then added for 30 min at room temperature in a wet chamber. The revelation was performed using two 3,3′-diaminobenzidine (DAB) chromogen incubations for 5 min each (Empire Genomics, Buffalo, NY, USA) at room temperature in a wet chamber. Harris’s hematoxylin staining (Intermedico, Markham, ON, Canada) followed by a dehydration of samples was performed before slides’ assembly. All washes were done using 1× TBS. Slides were left to dry for 24 h horizontally before analyses.

### 4.4. Interpretation of Anti-SFRP1 Immunostaining

Expression was assessed using a light microscope (Figure 1). Because of the absence of an existing standardized method for the quantification of the expression of SFRP1 by IHC, and the absence of evident relationship between its expression in a specific cell type and the degree of lobular involution, several methods were used and compared to classify the population study. First, the presence of staining (score = 1) vs. the total absence of staining (score = 0) was considered as a binary score of SFRP1 expression and obtained regardless of the cell types. Then, 17 elements were evaluated for the presence of staining (score = 1) vs. the total absence of staining (score = 0) to distinguish which elements of the tissue express the protein of interest and if there was any switch of expression during the timeline of lobular involution. The 17 elements considered were: The acini (epithelial cells nucleus, cytoplasm, luminal membrane, and the lumen), the ducts (epithelial cells nucleus, cytoplasm, luminal membrane, myoepithelial cells, lumen and circulating macrophages, and monocytes), the stroma, the adipose tissue (adipocytes’ nucleus and cytoplasm), blood vessels (endothelial and red blood cells), and the immune system cells (lymphocytes and macrophages). Then, a continuous score was obtained by adding scores of the evaluated elements for each patient, regardless of staining intensity. These scores were also divided into three categories: Total absence of SFRP1 expression (score = 0), average SFRP1 expression (scores = 1 to 5), and high SFRP1 expression (score > 5). Acini scores were also considered alone, but since results were similar to the continuous score of SFRP1 expression (rho = 0.79; *p* = 2.3 × 10^−16^) they are not presented. All lacking elements were treated either as missing values or replaced by a score of zero, without any impact on the final results. Two patients were excluded because of the absence of interpretable cores. The final cohort included 162 patients. To attest to the reproducibility of this scoring, 10% of the cores, i.e., two TMAs’ slides, were randomly selected and were read by an independent pathologist and the reader herself (rho = 0.70; *p*-value = 8.1 × 10^−13^ and rho = 0.93; *p*-value = 2.2 × 10^−16^, respectively). 

### 4.5. Inflammatory Markers’ Immunostaining and Interpretation

Selection and IHC staining methods of inflammatory markers were detailed in a previous study by Hanna et al. [15]. Briefly, anti-IL-6 (mouse monoclonal antibody (mAb), sc-130326; Santa Cruz Biotechnology, Santa Cruz, CA, USA, 1:150 for 1 h), anti-TNF-α (mouse mAb, (52B83); Santa Cruz Biotechnology, Santa Cruz, CA, USA, 1:50 for 2 h), anti-CRP (rabbit mAb, 1568–1; Epitomics, Burlingame, CA, USA, 1:100 for 1 h), anti-COX-2 (mouse mAb, 358200; Invitrogen, La Jolla, CA, USA, 1:100 for 1 h), anti-Leptin (rabbit polyclonal antibody (pAb), (A-20) sc842; Santa Cruz Biotechnology, Santa Cruz, CA, USA, 1:200 for 1 h), anti-SAA1 (mouse mAb, AT375a; ABGENT, San Diego, CA, USA, 1:50 for 1 h), anti-phospho-STAT3 (rabbit mAb, 2236–1; Epitomics, Burlingame, CA, USA, 1:250 overnight incubation), anti-IL-8 (mouse mAb, 60141-1-Ig; Proteintech Group, Rosemont, IL, USA, 1:100 for 2 h), anti-TGF-β (mouse mAb, MCA797; AbDserotec, Kidlington, UK,, 1:500 for 1 h), anti-IL-10 (rat mAb, MCA2250; AbDserotec, Kidlington, UK, 1:200 overnight incubation), and anti-lactoferrin (rabbit mAb, 3271–1; Epitomics, Burlingame, CA, USA, 1:100 for 1 h) were used after heat-induced epitope antigen retrieval using prewarmed citrate buffer (pH = 6) for 12 min for COX-2, SAA1, STAT3, IL-8, TGF-β, IL-10, and lactoferrin stained slides. Corresponding secondary antibodies were used, either the anti-mouse antibody (DAKO, EnVision+ System-HRP (DAB), Bucks, UK) or the rat, rabbit, and guinea pig antibody (IDetect Super Stain HRP Polymer Kit, ON, Canada). Staining intensity was scored 0–3 corresponding to negative, weak, medium, or strong staining, respectively. The extent of staining was expressed as the proportion of positively stained cells, scoring 0, 1, 2, or 3 for 0%, 1–9%, 10–50%, and >50% of cells stained positive, respectively. The immunostaining was individually evaluated in each of the 1–6 cores judged interpretable on the TMA-stained slides for each woman. Then, the median of the intensity and the extent of all cores were estimated. Next, the quick score was obtained by multiplying the median of the intensity (0–3) by that of the extent (0–3) and then dichotomized into low vs. high expression using the median of each marker as a cutoff. 

### 4.6. RNA Isolation and Quantitative Real-Time PCR (qRT-PCR)

RNA isolation and qRT-PCR are detailed in a previous study by Slim et al. [32]. Briefly, samples of formalin-fixed and paraffin-embedded (FFPE) breast adipose tissue from breast cancer patients were extracted with miRNeasy (FFPE) kit (Qiagen, Toronto, ON, Canada according to manufacturer’s instructions. RNA was reverse-transcribed using the Superscript IV kit (Invitrogen, La Jolla, CA, USA) according to manufacturer’s instructions. Oligoprimer pairs were designed by GeneTool 2.0 software (Biotools Inc, Edmonton, AB, Canada) and their specificity was verified by blast in the GenBank database. The synthesis was performed by IDT (Integrated DNA Technology, Coralville, IA, USA; Table S3). The cDNA corresponding to 20 ng of total RNA was used to perform fluorescent-based real-time PCR quantification using the LightCycler 480 (Roche Diagnostics, Mannheim, DE, USA). LightCycler 480 SYBRGreen I Master reagent (Roche Diagnostics, Indianapolis, IN, USA) was used with 2% DMSO, as described by the manufacturer. PCR was carried out using the following parameters: 45 cycles, denaturation at 98 °C for 10 s, annealing at 60 °C for 10 s, and elongation at 72 °C for 10 s and then 72 °C for 5 s (reading). A melting curve analysis was performed to assess nonspecific signals. Relative quantity was calculated using the fit point method and by applying the delta cycle threshold (Ct) method [33]. ATP synthase subunit O (ATP5O), glucose-6-phosphate dehydrogenase (G6PD), hypoxanthine guanine phosphoribosyl transferase 1 (HPRT1), and glyceraldehyde-3-phosphate dehydrogenase (GAPDH) were used as reference genes. The qRT-PCR measurements were performed by the Centre Hospitalier Universitaire de Québec Research Center Gene Expression Platform, Quebec, Canada, and were compliant with MIQE guidelines [34,35].

### 4.7. Statistical Analyses

All analyses described were performed with *RStudio v1.2.5033* (RStudio Team (2019), RStudio: Integrated Development for R, RStudio, Inc., Boston, MA, URL http://www.rstudio.com/). Comparisons of means between two groups were performed using a t test for independent samples, with the *t*-test function of the package stats v3.6.2 (https://www.rdocumentation.org/packages/stats). Hypothesis testing, both univariate and multivariate, was performed by generalized linear model of regression with the glm function of the R package stats v3.6.2. Logistic regression (binomial model) was used to predict binary outcome (complete involution vs. no or partial involution) from a set of continuous predictive variables (SFRP1 expression, age at mastectomy). Correlation tests between quantitative variables (SFRP1 expression vs. leptin expression) were performed using the nonparametric Spearman method of the cor.test function of the stats v3.6.2. R package. The ROC curves were drawn, and the AUC obtained with the roc and plot.roc functions of the pROC v1.16.1 (https://www.rdocumentation.org/packages/pROC) R package. The *p*-values lower than 0.05 were considered as significant.

## 5. Conclusions

To conclude, SFRP1 expression in nontumoral breast tissue seems to be a good predictor of the degree of lobular involution in both premenopausal and postmenopausal women as well as a good predictor of microcalcifications’ development especially in nulliparous women. Furthermore, the higher expression of SFRP1 in nontumoral tissue of nulliparous women having microcalcifications suggests the existence of a cross talk between parity history, age-related lobular involution, and breast microcalcifications’ development. Our findings warrant further investigation of both these elements as determinant of breast tumorigenesis that could be crucial for the development of personalized breast cancer treatments.

## Figures and Tables

**Table 1:** Characteristics of the study population.

Characteristics	All (*n* = 162)	Premenopausal (*n* = 82)	Postmenopausal (*n* = 80)	*p*-Value
Age at mastectomy (years)	52 ± 7.9	47 ± 5.8	58 ± 4.9	<0.0001
Degree of lobular involution (*n*)				
No or partial involution	77 (48%)	51 (62%)	26 (32%)	0.00012
Complete involution	85 (52%)	31 (38%)	54 (68%)	
Predominant lobule type (*n*)				
Predominant type 1 without any type 3	65 (40%)	16 (20%)	49 (61%)	<0.0001
Predominant type 2–3	97 (60%)	66 (80%)	31 (39%)	
Microcalcifications (*n*)	67 (41%)	29 (35%)	38 (48%)	0.12
Waist circumference (cm)	87 ± 13	84 ± 12	90 ± 13	0.0018
Parity				
Nulliparous	44 (27%)	23 (28%)	21 (26%)	0.80
Parous	118 (73%)	59 (72%)	59 (74%)	
Number of deliveries * (mean)	2.1 ± 0.80	2.1 ± 0.81	2.0 ± 0.80	0.36
Age at the first delivery * (years)	26 ± 4.1	26 ± 4.3	26 ± 3.8	0.44
Breastfeeding * (*n*)	61 (52%)	33 (56%)	28 (47%)	0.49
Total breastfeeding duration * (months)	4.6 ± 7	5.5 ± 8.3	3.6 ± 5.3	0.13

* In parous women; continuous numbers are presented as mean ± standard deviation (SD), *p*-value < 0.05 is considered significant.

**Table 2:** Association between the expression of SFRP1 (continuous variable) and the degree of lobular involution.

Continuous Scores	All (*n* = 162)		Premenopausal (*n* = 82)		Postmenopausal (*n* = 80)	
	OR (95% CI)	*p*-Value	OR (95% CI)	*p*-Value	OR (95% CI)	*p*-Value
SFRP1 expression *	0.85 (0.76–0.95)	0.0053	0.80 (0.65–0.96)	0.027	0.85 (0.72–0.99)	0.036
SFRP1 expression * adjusted for age at mastectomy	0.83 (0.74–0.94)	0.0031	0.80 (0.65–0.97)	0.030	0.83 (0.70–0.98)	0.030
SFRP1 expression * adjusted for menopausal status	0.83 (0.73–0.93)	0.0024				
SFRP1 expression * adjusted for age at mastectomy, waist circumference and presence microcalcifications	0.83 (0.73–0.94)	0.0047	0.83 (0.73–0.94)	0.049	0.82 (0.66–0.99)	0.011

Abbreviations: OR, odds ratio; CI, confidence interval; SFRP1, secreted frizzled-related protein 1, *p*-value < 0.05 is considered significant. * Assessed in nontumoral tissue.

**Table 3:** Spearman correlation between SFRP1 expression, waist circumference, and inflammatory cytokines’ expression by epithelial cells and adipocytes in all women.

Continuous Variables	Cytokines	Epithelial Cells			Adipocytes		
		N	Rho	*p*-Value	N	Rho	*p*-Value
SFRP1	Pro-inflammatory markers:						
	Leptin	155	0.32	<0.0001	72	0.043	0.72
	COX-2	156	−0.060	0.45		-	-
	CRP	158	0.14	0.090		-	-
	SAA1	152	0.15	0.070		-	-
	STAT3	156	0.038	0.64		-	-
	TNF-α	157	0.21	0.007	62	−0.078	0.54
	IL-6	156	0.21	0.009	72	0.041	0.73
	IL-8	157	0.040	0.64		-	-
	Anti-inflammatory markers:						
	Lactoferrin	152	0.070	0.36		-	-
	IL-10	148	0.040	0.67		-	-
	TGF-β	158	−0.024	0.76		-	-
Waist circumference	Pro-inflammatory markers:						
	Leptin	155	−0.16	0.046	72	0.45	<0.0001
	COX-2	156	−0.080	0.31		-	-
	CRP	158	−0.22	0.0060		-	-
	SAA1	152	0.036	0.66		-	-
	STAT3	156	0.087	0.28		-	-
	TNF-α	157	−0.26	0.00091	62	0.16	0.21
	IL-6	156	−0.25	0.0016	72	0.37	0.001
	IL-8	157	−0.045	0.57		-	-
	Anti-inflammatory markers:						
	Lactoferrin	152	−0.070	0.37		-	-
	IL-10	148	−0.11	0.20		-	-
	TGF-β	158	−0.0061	0.94		-	-

Abbreviations: SFRP1, secreted frizzled-related protein 1; IL-8, interleukin 8; IL-6, interleukin 6; TNF-α, tumor necrosis factor α; COX-2, cyclooxygenase 2; SAA1, serum amyloid A1; STAT3, signal transducer and activator of transcription 3; CRP, C-reactive protein; TGF-β, transforming growth factor β; IL-10, interleukin 10. The *p*-value < 0.05 is considered significant.

**Table 4:** Inflammatory profile of breast tissue according to the degree of lobular involution, stratified by parity status.

Continuous Scores		Parous (*n* = 118)				Nulliparous (*n* = 44)	
		Breastfeeding (*n* = 61)		Without Breastfeeding (*n* = 57)			
		Involuted (*n* = 28)	Non-Involuted (*n* = 33)	Involuted (*n* = 38)	Non-Involuted (*n* = 19)	Involuted (*n* = 19)	Non-Involuted (*n* = 25)
SFRP1	Mean	1.9	3.1	2.2	3.1	1.1	3.3
	*p*-value	0.10		0.27		0.0030	
Pro-inflammatory markers							
IL-8 epithelium	Mean	3.0	3.7	3.3	3.3	2.8	3.4
	*p*-value	0.0083		0.98		0.019	
IL-6 epithelium	Mean	1.6	4.0	2.06	3.6	2.3	3.6
	*p*-value	0.0013		0.037		0.11	
Leptin epithelium	Mean	3.7	6.2	3.5	5.7	3.9	6.4
	*p*-value	0.00045		0.0067		0.0030	
TNF-α epithelium	Mean	1.6	3.8	1.7	3.4	1.7	4.1
	*p*-value	0.0011		0.021		0.0022	
COX-2 epithelium	Mean	4.8	6.0	4.6	5.7	4.4	6.1
	*p*-value	0.12		0.050		0.041	
COX-2 stroma	Mean	1.6	2.1	1.8	2.7	1.3	2.0
	*p*-value	0.35		0.044		0.090	
SAA1 epithelium	Mean	2.8	3.3	3.2	4.2	2.8	3.7
	*p*-value	0.17		0.11		0.033	
STAT3 epithelium	Mean	4.2	4.0	4.3	5.1	3.7	4.2
	*p*-value	0.76		0.20		0.51	
CRP epithelium	Mean	1.8	4.3	1.6	2.6	1.2	2.8
	*p*-value	0.00011		0.070		0.0070	
Anti-inflammatory markers							
TGF-β epithelium	Mean	1.1	2.2	2.0	1.8	1.7	1.3
	*p*-value	0.090		0.81		0.48	
IL-10 epithelium	Mean	3.5	5.0	4.3	5.3	3.3	5.4
	*p*-value	0.0075		0.14		0.0021	
Lactoferrin epithelium	Mean	4.0	4.5	4.1	4.8	2.4	4.7
	*p*-value	0.47		0.42		0.0062	

Abbreviations: MC, microcalcifications; SFRP1, secreted frizzled-related protein 1; IL-8, interleukin 8; IL-6, interleukin 6; TNF-α, tumor necrosis factor α; COX-2, cyclooxygenase 2; SAA1, serum amyloid A1; STAT3, signal transducer and activator of transcription 3; CRP, C-reactive protein; TGF-β, transforming growth factor β; IL-10, interleukin 10. The *p*-value < 0.05 is considered significant.

**Table 5:** Association between breast microcalcifications’ development and risk factors in all women (*n* = 162).

Characteristics	*T*-Test: Mean Comparison			Generalized Linear Model	
	MC	No MC	*p*-Value	OR (95% CI)	*p*-Value
Parous women (*n* = 118)					
Age at the first delivery (years)	27 ± 4.4	25 ± 3.5	0.0019	1.2 (1.1–1.3)	0.0029
Number of deliveries (*n*)	1.8 ± 0.69	2.3 ± 0.83	0.0033	0.63 (0.34–1.1)	0.12
Time of breastfeeding (month)	3.2 ± 5.3	5.5 ± 7.9	0.05	0.95 (0.87–1.0)	0.14
Postmenopausal (%)	44	56	0.47	1.3 (0.64–2.8)	0.45
SFRP1 expression (continuous)	2.9 ± 3.1	2.3 ± 2.9	0.31	1.1 (0.94–1.2)	0.30
Nulliparous women (*n* = 44)					
Postmenopausal (%)	57	43	0.080	3.1 (0.9–11)	0.080
SFRP1 expression (continuous)	3.7 ± 3.1	1.3 ± 1.8	0.0057	1.5 (1.1–2.0)	0.0075

Abbreviations: MC, microcalcifications; OR, odds ratio; CI, confidence interval; SFRP1, secreted frizzled-related protein 1. Continuous numbers are presented mean ± standard deviation (SD). The *p*-value < 0.05 is considered significant.

**Table 6:** Inflammatory profile of breast tissue in presence of microcalcifications, stratified for parity status in all women (*n* = 162).

Continuous Scores		Parous (*n* = 118)				Nulliparous (*n* = 44)	
		Breastfeeding (*n* = 61)		Without Breastfeeding (*n* = 57)			
		No MC (*n* = 37)	MC (*n* = 24)	No MC (*n* = 33)	MC (*n* = 24)	No MC (*n* = 25)	MC (*n* = 19)
SFRP1	Mean	2.7	2.3	1.9	3.4	1.3	3.7
	*p*-value	0.63		0.061		0.0057	
Pro-inflammatory markers							
IL-8 epithelium	Mean	3.4	3.4	3.3	3.3	2.9	3.4
	*p*-value	0.75		0.90		0.15	
IL-6 epithelium	Mean	3.0	2.9	2.3	3.0	3.2	2.8
	*p*-value	0.90		0.37		0.64	
Leptin epithelium	Mean	5.2	5.0	4.1	4.4	5.4	5.4
	*p*-value	0.80		0.71		0.97	
TNF-α epithelium	Mean	3.2	2.4	2.1	2.5	3.2	3.0
	*p*-value	0.24		0.49		0.79	
COX-2 epithelium	Mean	5.6	5.2	5.2	4.6	6.0	4.6
	*p*-value	0.55		0.30		0.09	
COX-2 stroma	Mean	2.3	1.2	2.0	2.2	1.8	1.6
	*p*-value	0.0085		0.63		0.48	
SAA1 epithelium	Mean	3.2	2.9	3.6	3.4	3.0	3.8
	*p*-value	0.27		0.74		0.12	
STAT3 epithelium	Mean	4.0	4.2	4.4	4.7	4.1	3.8
	*p*-value	0.73		0.64		0.68	
CRP epithelium	Mean	3.3	3.1	1.7	2.3	2.7	1.3
	*p*-value	0.87		0.27		0.019	
Anti-inflammatory markers							
TGF-β epithelium	Mean	1.9	1.4	1.3	2.8	1.8	1.1
	*p*-value	0.50		0.025		0.20	
IL-10 epithelium	Mean	4.2	4.4	4.9	4.4	4.6	4.6
	*p*-value	0.69		0.49		0.97	
Lactoferrin epithelium	Mean	4.4	4.1	4.5	4.1	4.2	3.1
	*p*-value	0.71		0.65		0.21	

Abbreviations: MC, microcalcifications; SFRP1, secreted frizzled-related protein 1; IL-8, interleukin 8; IL-6, interleukin 6; TNF-α, tumor necrosis factor α; COX-2, cyclooxygenase 2; SAA1, serum amyloid A1; STAT3, signal transducer and activator of transcription 3; CRP, C-reactive Protein; TGF-β, transforming growth factor β; IL-10, interleukin 10. The *p*-value < 0.05 is considered significant.

## Supplementary Materials

The following are available online at https://www.mdpi.com/2072-6694/12/9/2693/s1, Table S1: Association between SFRP1 expression (binary variable) and the degree of lobular involution, Table S2: Histopathological characteristics of the study population, Table S3: Primer sequences and gene description.
