# Supplementary material for: Secreted Frizzled-Related Protein 1 as a Biomarker against Incomplete Age-Related Lobular Involution and Microcalcifications’ Development
Source: Cancers (Basel). 2020 Sep 21;12(9):2693. doi: 10.3390/cancers12092693 (PMC7565692; doi:10.3390/cancers12092693)
Supplement: Supplementary file 1 [file cancers-12-02693-s001.pdf]

cancers

MDPI

Table S1. Association between SFRP1 expression (binary variable) and the degree of lobular involution.

Binary scores	All (n = 162)		Premenopausal (n = 82)		Postmenopausal (n = 80)	
	OR (95% CI)	*p*-value	OR (95% CI)	*p*-value	OR (95% CI)	*p*-value
SFRP1 expression	0.28 (0.14–0.55)	0.00024	0.17 (0.060–0.45)	0.00048	0.44 (0.16–1.2)	0.11
SFRP1 expression adjusted for age at mastectomy	0.25 (0.12–0.50)	0.00018	0.18 (0.06–0.47)	0.00068	0.35 (0.10–0.99)	0.0080
SFRP1 expression adjusted for menopausal status	0.27 (0.13–0.54)	0.00028				
SFRP1 expression adjusted for age at mastectomy, waist circumference and presence of microcalcifications	0.25 (0.11–0.52)	0.00030	0.17 (0.060–0.48)	0.00098	0.30 (0.09–0.92)	0.044

Abbreviations: OR, odds ratio; CI, confidence interval; SFRP1, secreted frizzled-related protein 1. The *p*-value < 0.05 is considered significant.

Table S2. Histopathological characteristics of the study population.

Characteristics	All (n = 162)	Premenopausal (n = 82)	Postmenopausal (n = 80)
Histological type			
Ductal, in-situ	16 (10%)	9 (11%)	7 (9%)
Ductal, invasive	121 (75%)	67 (82%)	54 (67%)
Lobular, invasive	15 (9%)	4 (5%)	11 (14%)
Others *	10 (6%)	2 (2%)	8 (10%)
Tumor grade			
Non-assessed	26 (16%)	11 (13%)	15 (19%)
I	29 (18%)	15 (18%)	14 (17%)
II	65 (40%)	35 (43%)	30 (38%)
III	42 (26%)	21 (26%)	21 (26%)
ER status			
Positive	146 (90%)	75 (91%)	71 (89%)
Negative	16 (10%)	7 (9%)	9 (11%)
PR status			
Positive	136 (84%)	75 (91%)	61 (76%)
Negative	26 (16%)	7 (9%)	19 (24%)
HER2 status			
Not evaluated	28 (17%)	11 (13%)	17 (21%)
Positive	18 (11%)	8 (10%)	10 (13%)
Negative	116 (72%)	63 (77%)	53 (66%)

ER = estrogen receptor, PR = progesterone receptor, HER2 = human epidermal growth factor 2. * Includes mucinous, tubular, adenoid cystic, and metaplastic carcinomas.

Table S3. Primer sequences and gene description.

Gene Symbol	Description	GenBank	Size (pb)	Primer Sequence 5′→3′ Sens/Antisens
HPRT1	Homo sapiens hypoxanthine phosphoribosyltransferase 1	NM_000194	157	AGTTCTGTGGCCATCTGCTTAGTAG/AA ACAACAATCCGCCCAAAGG
GAPDH	Homo sapiens glyceraldehyde-3-phosphate dehydrogenase	NM_002046	194	GGCTCTCCAGAACATCATCCCT/ACGCC TGCTTCACCACCTTCTT
ADNg	Homo sapiens 3-beta-hydroxysteroid dehydrogenase/delta-5-delta-4-isomerase (3beta-HSD) gene (intron)	M38180	260	GAAGGGCAGAGGTGGAACTAGAA/AAC AAAGACCAAAGACCAGTGAGA
Leptin	Homo sapiens leptin (LEP)	NM_000230	64	TTTTGTCAAGTGTCATATGTAGGTGTC/CTCCCTTCTGCCCAAACATTC
IL-6	Homo sapiens interleukin 6 (interferon, beta 2)	NM_000600	70	CTGGTGTTGCCTGCTGCCTTC/GTGGGG CGGCTACATCTTTGG
TNFα	Homo sapiens tumor necrosis factor	NM_000594	85	CATCAAGAGCCCCTGCCAGAG/GAAGA CCCCTCCCAGATAGATG
ATP5O	Homo sapiens ATP synthase, H+ transporting, mitochondrial F1 complex, O subunit	NM_001697	103	ATTGAAGGTCGCTATGCCACAG/CCTTC AGGATTTGTGCTACTCTCA
G6PD	Homo sapiens glucose-6-phosphate dehydrogenase (G6PD), nuclear gene encoding mitochondrial protein	NM_000402	77	GCCAACCGCCTCTTCTACCTG/ATGCAG GACTCGTGAATGTTCTTG
